# Brazilian Consumer Attitude Towards the Concept of Meat Products with Claims of Naturalness, Healthiness and Sustainability

**DOI:** 10.3390/foods15030572

**Published:** 2026-02-05

**Authors:** Hellencris Cassin Rocha, Sabrina Souza França, Danielle Rodrigues Magalhães, Alessandra Lopes de Oliveira, Marco Antonio Trindade

**Affiliations:** Department of Food Engineering, Universidade de Sao Paulo Faculdade de Zootecnia e Engenharia de Alimentos, Pirassununga, São Paulo 13635-900, Brazil; hellencassin@usp.br (H.C.R.); sabrina.souza.franca@usp.br (S.S.F.); d.magalhaes@usp.br (D.R.M.); alelopes@usp.br (A.L.d.O.)

**Keywords:** agro-industrial by-products, food innovation, natural antioxidants, synthetic antioxidants

## Abstract

This study investigated Brazilian consumers’ perceptions, attitudes, and purchase intentions regarding traditional and reformulated chicken meat products (fresh sausage and burger) enriched with natural antioxidants obtained from avocado by-product extracts. A mixed-methods approach was applied, using a word association task, a Likert-scale attitudinal questionnaire, and purchase intention scales (*n* = 422). Word association revealed predominantly negative perceptions toward products containing synthetic antioxidants, while natural antioxidant formulations elicited positive associations related to health, naturalness, and sustainability. Attitudinal data indicated strong alignment between health consciousness, environmental concern, and openness to food innovation. Pearson correlations (*p* < 0.05) showed moderate-to-strong relationships (r ≥ 0.40) among beliefs about healthy eating, perceived benefits of natural antioxidants, and support for sustainable production. Contingency analyses demonstrated that belief in the health benefits of natural antioxidants significantly increased purchase intention for reformulated products, whereas consumers less engaged with healthy eating were more accepting of synthetic formulations. Noting sample limitations primarily comprising young, educated females, who correspond to the group of consumers who tend to be more sensitive to health and environmental responsibility claims, the findings highlight consumer interest in natural, functional, and sustainable meat products. These results reinforce the potential of using agro-industrial by-product extracts as natural antioxidants in meat formulations and underscore the importance of communication strategies emphasizing health, naturalness, and sustainability to improve consumer acceptance.

## 1. Introduction

Understanding consumer perceptions and behavior is essential for the development of food products that achieve market acceptance [1]. The cognitive processing of information about a food product occurs in three stages: exposure, attention, and interpretation. During these stages, sensory stimuli are initially received, then filtered according to internal goals or expectations, and finally interpreted based on the consumer’s personal knowledge and past experiences. Therefore, the concept of “quality” is subjective and shaped by individual needs and expectations [2].

Food choices are influenced by multiple factors and their interactions, including social, cultural, psychological, nutritional, environmental, and convenience-related aspects [3]. With regard to meat consumption patterns over time, perceptions of naturalness and healthiness, as well as the presence of clean labels indicating the absence of artificial ingredients and the inclusion of eco-score seals, have become increasingly important drivers of consumer purchase decisions [4,5]. Previous studies evaluating Brazilian consumers’ perceptions of reformulated meat products have shown a growing interest in healthier meat options; however, acceptance remains strongly conditioned by sensory expectations, level of information, and degree of health concern [6,7].

Furthermore, factors such as animal welfare and the environmental impact associated with meat production systems have emerged as important motivators of consumer attitudes, although sensory attributes and affordable prices remain central to product acceptance [8]. In addition, mass media and social networks have played a significant role in disseminating information about meat production and consumption, particularly regarding naturalness, health, and sustainability, thereby influencing consumer perceptions and behavior [8].

Processed meat products are those in which raw materials undergo specific technological transformations aimed at improving sensory characteristics, ensuring safety, and extending shelf life. Examples include hamburgers, sausages, and frankfurters, among others [9]. The consumption of processed meat is often perceived ambivalently, being regarded as both beneficial and harmful to human health [10]. The main benefits are associated with nutritional value and safety, as meat is well recognized for its high biological-value nutrients [11], while processing contributes to microbiological safety and the preservation of sensory quality [12]. Conversely, health concerns are related to the high fat content of certain processed meats and the use of chemically synthesized substances intended to inhibit oxidation and microbial growth, which, at high intake levels, may pose health risks [13,14].

In this context, alternatives aimed at producing healthier meat products have gained increasing attention. Many natural raw materials, particularly agro-industrial residues such as peels, seeds, and leaves, are rich in bioactive compounds with strong antioxidant and antimicrobial properties [15]. As an example, avocado is a fruit widely recognized for its health benefits and can be consumed in various forms with multiple commercial applications [16]. According to data from the Brazilian Institute of Geography and Statistics, Brazil produced 427,457 tons of avocados in 2024, ranking as the seventh largest producer worldwide [17]. However, approximately 30% of the fresh avocado weight corresponds to peels and seeds, which are discarded as by-products during fruit processing [16]. Therefore, incorporating these raw materials into food formulations represents both a strategy for developing healthier products and an environmentally responsible approach, as it promotes the valorization of residues that would otherwise require costly disposal processes [18,19], as well as helping to meet consumer expectations regarding natural, healthy, and sustainable attributes. From a behavioral perspective, the acceptance of these reformulated products can be understood in light of the Theory of Planned Behavior (TPB), which posits that consumer intentions are influenced by attitudes toward behavior, perceived social norms, and perceived behavioral control [20].

Previous studies have shown that attributes such as perceived naturalness, clean labels, and environmental responsibility exert a positive influence on consumer perception and purchase intention toward meat products [1], thereby increasing interest in reformulated products carrying these appeals [5,8]. However, despite this favorable attitudinal context, the literature also highlights the persistence of discrepancies between positive attitudes and actual purchasing behavior, which are often moderated by factors such as price, convenience, familiarity, and sensory perception [11]. Moreover, although progress has been made in understanding consumer acceptance of meat products with functional or clean-label claims, studies that integratively examine how consumers simultaneously combine attributes of naturalness, healthiness, and sustainability remain limited, particularly when these attributes are linked to the valorization of agro-industrial by-products as sources of natural antioxidants. This gap is especially evident in the Brazilian context, where empirical evidence based on mixed-method approaches exploring perceptions, attitudes, and purchase intentions toward reformulated meat products remains scarce. Addressing this gap is essential to support product development strategies aligned with consumer expectations and the transition toward more sustainable food systems.

Thus, it is hypothesized that reformulating meat products toward greater naturalness, such as replacing chemically synthesized additives with natural alternatives, particularly those derived from agro-industrial by-products, may help meet consumer expectations regarding natural, healthy, and sustainable attributes. Accordingly, this study aims, through a mixed-methods approach combining qualitative and quantitative consumer research techniques, to investigate Brazilian consumers’ perceptions of chicken sausages and hamburgers containing avocado peel and seed extracts as natural antioxidants, as well as to explore consumer behavior regarding factors that stimulate or limit the acceptance of both traditional and innovative products.

## 2. Materials and Methods

### 2.1. Study Design and Participants

To gain insights into consumer perceptions of traditional meat products (fresh chicken sausage and chicken burgers formulated with the synthetic antioxidant sodium erythorbate) and innovative products (with health and sustainability appeals resulting from the addition of avocado by-product extracts), an online questionnaire was developed using Google Forms (Google Web Platform). To recruit participants, the questionnaire was distributed via email and social media and was available for responses from January to June 2025 and was completed by 422 participants (*n* = 422). Two non-probabilistic sampling techniques were employed, convenience sampling and snowball sampling, with the aim of recruiting, according to predefined criteria, regular consumers of meat products [21,22,23].

Among the methods used to assess consumer behavior in food product development, qualitative approaches are applied to explore and understand motivations, emotions, and perceptions that cannot be fully captured by quantitative methods [24], whereas quantitative approaches measure and quantify variables in an objective and statistical manner, allowing results to be generalized to a broader population [25]. The combination of both approaches enables the integration of the depth of qualitative insights, capturing emotions and attitudes, with the breadth of quantitative data, facilitating the identification of consumption patterns and behavioral trends.

The questionnaire collected socioeconomic and demographic information to determine the frequency and percentage distribution of participant profiles, in addition to three methodological approaches: (i) a projective word association technique; (ii) purchase intention assessment; and (iii) an attitudinal questionnaire consisting of closed-ended statements evaluated using a 5-point Likert scale, ranging from 1 (strongly disagree) to 5 (strongly agree). The study was approved by the Research Ethics Committee of FZEA/USP on 3 December 2024, under protocol number 84067724.6.0000.5422, and all participants provided informed consent. The products used as stimuli in the study were:

• Traditional fresh chicken sausage (stimulus A);
• Fresh chicken sausage with avocado peel and seed extracts (stimulus B);
• Traditional chicken hamburger (stimulus C);
• Chicken hamburger with avocado peel and seed extracts (stimulus D).

### 2.2. Methods and Data Analysis

#### 2.2.1. Projective Word-Association Technique

This technique aimed to capture unconscious perceptions and immediate associations elicited by meat products (fresh chicken sausage and chicken burgers) formulated with either synthetic or natural antioxidants. Participants were asked to report the first four images, associations, thoughts, or feelings that came to mind for each product formulated with different antioxidants [26].

#### 2.2.2. Attitudinal Questionnaire (5-Point Likert Scale)

This part of the study aimed to analyze consumer behavior in relation to health and sustainability. Participants indicated their level of agreement with different statements related to these themes using a 5-point scale ranging from 1 (strongly disagree) to 5 (strongly agree) [23,25]. The statements were selected and adapted from previous studies addressing consumer perceptions of foods with health- and sustainability-related attributes [5,6,7,27].

#### 2.2.3. Purchase Intention Assessment

This method aimed to evaluate participants’ purchase intention for each stimulus (A, B, C, or D) in relation to their attitudes toward food healthiness and adherence to a healthy diet. Using a 5-point purchase intention scale ranging from “certainly would not buy” to “certainly would buy,” participants selected the option that best represented their intention for each product (stimulus).

#### 2.2.4. Data Analysis

Based on the absolute frequency of terms provided in the projective word association task, a word cloud was generated to qualitatively visualize consumer perceptions of meat products formulated with synthetic antioxidants (sodium erythorbate) or natural antioxidants (avocado peel and seed extracts). Variations in the same word were grouped, and “stop words” were also removed. Terms that were mentioned 168 times or more, or 10% of all the words gathered using the projective technique, were counted to produce the word cloud employing WordArt software (web-based software; https://wordart.com, accessed on September 2025). Data from the attitudinal questionnaire were subjected to Pearson’s bivariate correlation analysis, considering positive correlations ≥ 0.40 as indicative of moderate strength. For statistical presentation purposes, the original 5-point agreement scale was recoded into a 3-point scale. Mean purchase intention scores for each product (stimulus) were analyzed using contingency analysis (χ^2^), and, for presentation purposes, the original 5-point scale was likewise recoded into a 3-point scale. All statistical analyses were performed using IBM^®^ SPSS Statistics, version 30.0.

## 3. Results and Discussion

### 3.1. Sociodemographic Profile of Participants

**Scheme 1:**
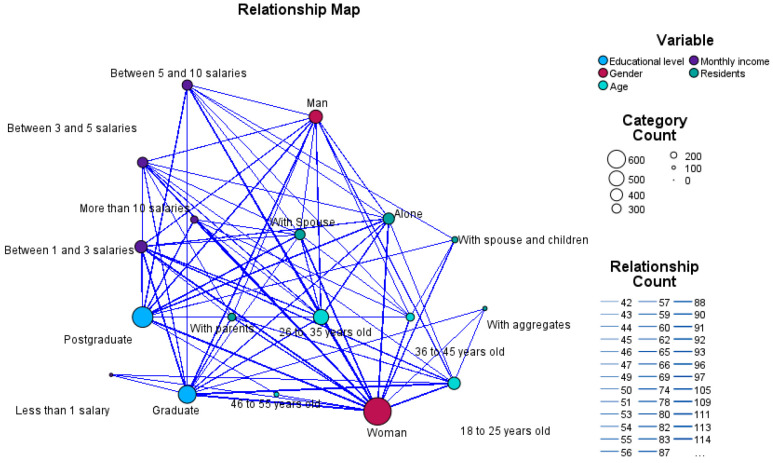
Relationship map illustrating the influence among sociodemographic variables in the studied population. Variables represented by larger nodes and thicker connecting lines indicate stronger influences, whereas smaller nodes and thinner lines indicate weaker influences. *n* = 422.

The sociodemographic profile of the studied population, as well as the associations among respondent profiles according to education level, gender, age, income, and place of residence, are presented in Table 1 and Scheme 1, respectively. The relationship map, which shows connections and influences through nodes, was made using SPSS software as a graphical display to look at how sociodemographic variables connected to one another. The sample was predominantly composed of women (69.7%), young adults aged between 18 and 35 years (67.0%), individuals with higher education levels (93.1% holding undergraduate or postgraduate degrees), and residents of the Southeast region of Brazil (88.6%). Household income was mainly distributed between 1 and 10 minimum wages (77.2%), indicating a predominantly middle-income profile. In Scheme 1, larger nodes and thicker connecting lines represent variables with greater influence within the network, indicating stronger associations among respondent profiles.

These findings indicate that the sample is predominantly composed of young women with university-level education, which is consistent with patterns reported in studies on consumer perceptions of food naturalness, healthiness, and sustainability, in which women and individuals with higher education levels tend to exhibit greater sensitivity to health- and environmental-responsibility claims [5,28,29]. In contrast, men with household incomes above ten minimum wages and individuals over 36 years of age showed lower representativeness and weaker connections with other variables. Overall, the observed patterns suggest greater interest in conscious consumption and health-related attributes among younger and digitally connected populations, a profile commonly identified in online surveys addressing innovative food products [26].

### 3.2. Projective Word-Association Technique

**Figure 1:**
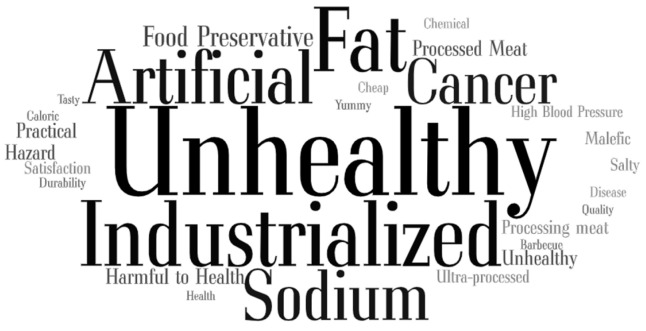
Word cloud illustrating consumer perceptions of chicken meat products formulated with synthetic antioxidants (sodium erythorbate).

**Figure 2:**
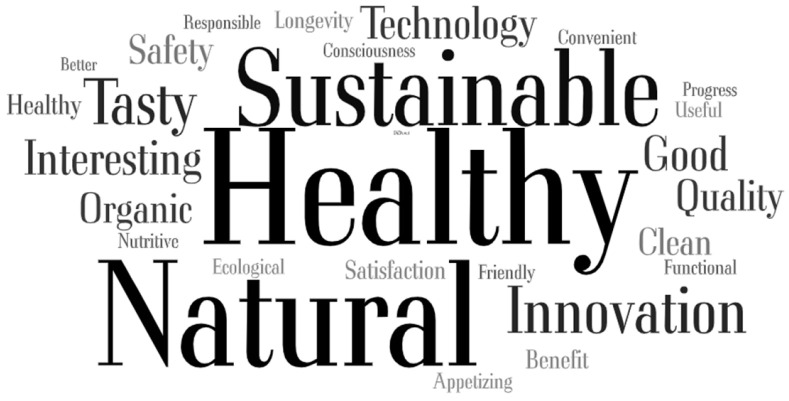
Word cloud representing consumer perceptions of chicken meat products formulated with natural antioxidants (avocado peel and seed extracts).

Figure 1 and Figure 2 present the word clouds generated using the projective word association technique, providing a qualitative visualization of consumer perceptions of meat products formulated with synthetic antioxidants (sodium erythorbate) and natural antioxidants (avocado peel and seed extracts), respectively. The size of each word corresponds to its frequency of mention.

In Figure 1, negative terms, in order of frequency, such as unhealthy, industrialized, fat, sodium, and artificial, reflecting associations related to perceived health risks and the presence of chemical additives. In contrast, Figure 2 shows a predominance of positive terms, including healthy, natural, sustainable, and innovation, indicating favorable associations with health, quality, and environmental responsibility.

The terms identified in Figure 1 indicate perceptions of risk and distrust toward the use of synthetic additives. These associations may be linked to perceptions of excessive processing and artificiality, which is consistent with previous studies reporting increasing consumer rejection of products perceived as “ultra-processed” [30,31]. The presence of terms such as cancer, hypertension, and disease reflects direct associations between synthetic additives and negative health outcomes, perceptions that are often influenced by media narratives and public debates surrounding healthy eating [5,8].

Conversely, Figure 2 highlights of positive associations, indicating consumer appreciation for attributes related to naturalness, functionality, and environmental responsibility. Terms such as technology, organic, and different reinforce a perception of sustainable innovation, aligning with contemporary trends in conscious consumption and preferences for clean-label foods [1,3].

This contrast demonstrates that the use of natural ingredients derived from agro-industrial by-products, beyond representing a sustainable technological strategy, also acts as a competitive advantage capable of improving consumer perceptions of meat products [11]. These findings reinforce the argument that perceived naturalness is one of the primary determinants of acceptance of reformulated foods, often outweighing attributes such as price and familiarity [2,29]. Therefore, communication strategies emphasizing the valorization of agro-industrial residues and the antioxidant and functional properties of natural extracts may enhance consumer trust and engagement with innovative meat products, promoting a transition toward more conscious and sustainable consumption practices [32].

### 3.3. Consumer Behavior Regarding Healthiness and Sustainability

The attitudinal questionnaire assessed consumer behavior toward health and sustainability based on the level of agreement with a set of statements. Response frequencies according to the Likert scale are presented in Table 2. Overall, the analysis reveals a positive trend in consumer engagement with foods carrying claims related to naturalness, health, and sustainability, as well as openness to innovative food proposals, despite the presence of certain behavioral contradictions.

Regarding openness to experimentation, statements QA1 and QA2 indicate that most participants (63.5% and 59%, respectively) are willing to try new foods, an essential factor for the acceptance of reformulated products containing alternative ingredients. This result is consistent with the sociodemographic profile shown in Figure 1, which highlights a predominance of young, highly educated women—a group generally more receptive to innovation and to products with natural, healthy, and sustainable appeal [28,29].

Statements QA3, QA4, and QA5, which address health-oriented attitudes, show that approximately half of the participants (50–53.6%) value healthy eating and are willing to choose healthier foods even at the expense of sensory pleasure. In addition, 75.6% of respondents agreed that antioxidants reduce disease risk (QA6), suggesting acceptance of natural antioxidants as substitutes for synthetic ones and reinforcing the credibility of functional claims associated with innovative formulations [1,4]. The high levels of agreement observed for QA7 (90%) and QA8 (66.8%) indicate strong purchase intentions linked to health-related values, even at higher price levels, which is consistent with the literature describing “health-oriented” consumers who associate higher prices with higher perceived quality [26,27].

Statements QA9 and QA10 reflect attitudes related to convenience: 37% of participants reported enjoying the consumption of practical foods even if they are less healthy, while 58% disagreed with the statement that they disregard health when consuming quick-preparation products. These findings highlight the contemporary consumer’s preference for convenience without fully compromising perceived naturalness, which is often associated with healthier food choices [31].

The sustainability dimension also showed expressive results. A total of 69.9% of participants considered sustainable production important (QA11), 53.1% reported being willing to pay more for such products (QA12), and 71.6% disagreed with the idea that sustainability “is not their responsibility” (QA13). In addition, 84.6% agreed that the agro-industry should minimize waste and environmental impacts (QA14), while 65.4% disagreed with the notion that sustainable food production is not a priority (QA16). Together, these results indicate consumer awareness of the environmental impacts associated with food production chains and a willingness to consume foods carrying sustainability claims, although practical limitations remain, as only 32% of participants considered it easy to purchase sustainable foods (QA15). Overall, these findings reveal a relevant consumer segment inclined toward purchasing products produced under sustainable systems, reinforcing the market potential for foods with sustainability-related appeals [33,34].

Previous research has identified an “attitude–behavior gap,” in which consumers express strong sustainability values but do not consistently translate them into purchasing behavior due to barriers such as cost, access, and convenience [5,29]. However, the results of the present study indicate a tendency toward narrowing this gap, as a substantial proportion of participants demonstrated a willingness to pay more for healthy and sustainable products and acknowledged both individual and collective responsibility in promoting responsible consumption. These findings suggest that, although external factors such as price and availability continue to act as barriers, positive attitudes toward health and sustainability are increasingly being reflected in purchase intentions [33,34].

The consumer profile observed in this study is also consistent with the Theory of Planned Behavior [20], which proposes that behavioral intentions are shaped by three main components: attitudes (positive or negative evaluations of a given behavior), subjective norms (perceived social pressure to perform or not perform the behavior), and perceived behavioral control (the perceived ease or difficulty of performing the behavior) [20,35]. High levels of agreement with statements related to naturalness, health, and sustainability reflect favorable attitudes toward the consumption of products with these attributes, while the expressed willingness and interest indicate strong motivation and internalization of pro-health and pro-environmental values [36]. At the same time, factors such as price, convenience, and accessibility represent dimensions of perceived behavioral control and continue to act as limiting factors [37]. In this context, limited access emerges as a key barrier to full adoption, as only 32% of participants reported that sustainably produced foods are easy to obtain. Nevertheless, the findings demonstrate that consumers exhibit openness to food innovation, positive perceptions of natural antioxidants, and pro-health and pro-sustainability attitudes, even in the presence of behavioral and economic constraints that may hinder widespread adoption of these products.

### 3.4. Correlations Among Health- and Sustainability-Related Statements

Table 3 presents the correlations among the attitudinal statements, revealing patterns of relationships between consumer attitudes toward innovation, healthiness, and sustainability. All correlations were statistically significant (*p* < 0.05), indicating meaningful associations among the constructs.

Moderate correlations were observed between QA3–QA5 (r = 0.498) and QA6–QA7 (r = 0.436), indicating consistency between self-perceived healthy eating, belief in the benefits of natural antioxidants, and willingness to consume foods with positive health impacts. Moderate correlations were also identified between QA9–QA10 (r = 0.431), suggesting that even health-conscious consumers continue to value convenience [26]. The correlation between QA7–QA11 (r = 0.513) indicates that individuals concerned with personal health also value sustainable production practices, reinforcing the contemporary concept of a “health–sustainability convergence” [1,5].

In addition, moderate to strong correlations were observed among sustainability-related variables, particularly: QA7–QA14 (r = 0.572), reflecting the relationship between willingness to consume healthy products and belief in the role of agribusiness in reducing environmental impacts; QA8–QA12 (r = 0.604), indicating an association between intention to consume health-beneficial products and willingness to pay more for sustainably produced foods; QA11–QA12 (r = 0.567), linking the perceived importance of sustainable production with willingness to pay more for sustainable products; and QA11–QA14 (r = 0.438), relating the importance of sustainable production to the belief that agribusiness should strive to minimize waste and environmental impacts.

Correlations among health-related statements indicate that consumers with healthier eating habits also tend to recognize and value functional ingredients, and that awareness of the effects of natural compounds represents an important predictor of the intention to purchase foods labeled as “clean label” [4]. Furthermore, correlations involving sustainability-related variables reinforce that consumers who demonstrate concern for personal well-being also tend to adopt attitudes aligned with environmentally responsible production practices. This pattern corroborates the contemporary trend of “health–sustainability convergence” [1,5], in which the sense of social and environmental responsibility associated with conscious purchasing intentions validates the profile of consumers who perceive ethical and sustainability values as extensions of personal health [8,29].

### 3.5. Purchase Intention for Products with Synthetic vs. Natural Antioxidants and Belief in the Health Benefits of Natural Antioxidants

The contingency analysis (χ^2^) presented in Table 4 shows significant associations (*p* < 0.05) between beliefs about natural antioxidants and purchase intention for sausages and hamburgers formulated with different types of antioxidants. These results indicate that cognitive attitudes related to perceived health benefits directly influence declared purchasing behavior.

For sausages formulated with synthetic antioxidants (SA), consumers who disagreed with the statement “consumption of natural antioxidants may reduce the risk of disease” exhibited higher purchase intention (55.6%) compared with those who were indifferent (40.8%) or agreed with the statement (29.5%). A similar pattern was observed for hamburgers with synthetic antioxidants, in which consumers skeptical about the benefits of natural antioxidants showed a greater propensity to purchase (55.6%) than those who believed in these benefits (30.6%). These findings suggest that skepticism toward natural compounds is associated with greater acceptance of traditional formulations, possibly due to factors such as consumption habits, familiarity, and trust in conventional food technologies [31].

Conversely, purchase intention was substantially higher among consumers who believed in the benefits of natural antioxidants. For sausages formulated with natural antioxidants, 69.6% of respondents who agreed with the statement indicated purchase intention, compared with 48.1% of those who disagreed and 57.9% of those who were indifferent. A similar trend was observed for hamburgers with natural antioxidants, with 67.7% of consumers who believed in the health benefits reporting purchase intention, a proportion notably higher than that observed among indifferent (57.9%) and disagreeing (51.9%) participants. These results reinforce that belief in the physiological benefits of natural compounds is a decisive driver of purchase intention for innovative products [1,4]. The alignment between pro-health attitudes and purchasing decisions supports the identification of a segment of conscious consumers who value naturalness and transparency in labeling, consistent with the clean-label trend.

The pattern observed across the four products highlights a dissociation between consumer groups: a *traditionalist* group, which demonstrates greater familiarity and comfort with synthetic additives and lower sensitivity to naturalness; and a *conscious and innovative* group, which recognizes the value of natural compounds and shows a greater intention to purchase products with health and sustainability appeals [38]. From a market segmentation perspective, these results indicate the coexistence of distinct consumer segments with different motivations and levels of engagement with food innovation, reinforcing that products with natural, health, and sustainability claims tend to appeal more strongly to consumers already aligned with these values [28,29].

Furthermore, the fact that all relationships were statistically significant (*p* < 0.05) confirms that health-related beliefs modulate both sensory and commercial acceptance of reformulated products, in agreement with the Theory of Planned Behavior [20] and the role of cognitive attitudes as predictors of purchase intention [28]. These results corroborate the literature indicating a growing trend toward valuing natural ingredients, particularly when consumers understand their benefits and perceive credibility in label information [5,8]. Conversely, the persistence of a substantial segment of indifferent consumers suggests the need for educational and communication strategies aimed at increasing public awareness of natural antioxidants and food sustainability concepts [34,39], which may support a gradual shift in these consumers toward more engaged segments.

### 3.6. Purchase Intention and Adherence to a Healthy Diet

The contingency analysis (χ^2^) presented in Table 5 revealed statistically significant relationships (*p* < 0.05) between purchase intention and the level of agreement with the statement “I follow a healthy and balanced diet” for three of the four products analyzed (all combinations except the hamburger formulated with a natural antioxidant, *p* = 0.068). These results indicate that self-perceived healthy eating behavior is associated with a willingness to try innovative products, particularly those with appeals related to naturalness and functionality.

For products formulated with synthetic antioxidants, consumers who disagreed with the statement “I follow a healthy diet” exhibited higher purchase intention (59.6% for sausages and 61.0% for hamburgers) compared with those who agreed with the statement (40.0% and 41.1%, respectively). This pattern suggests that individuals who are less concerned with health tend not to reject conventional products, possibly due to factors such as familiarity, habitual consumption, and convenience [8]. Conversely, consumers who reported following a healthy diet showed lower predisposition to purchase products containing synthetic antioxidants, reinforcing a more selective food awareness and highlighting perceived naturalness as a key attribute in food purchasing decisions [29,31].

For chicken sausages formulated with avocado extracts, the highest purchase intention (57.5%) was observed among consumers who were indifferent to adherence to a healthy diet, followed by those who agreed (48.7%) and disagreed (46.0%). This finding suggests that even among individuals who do not explicitly report healthy eating behaviors, products carrying natural claims may still achieve positive acceptance [40]. Regarding hamburgers formulated with natural antioxidants, although the association between variables did not reach statistical significance (*p* = 0.068), purchase intention showed a progressive increase according to the level of agreement with following a healthy diet (from 48.4% to 50.4%).

This trend reinforces the notion that health perception positively influences the acceptance of reformulated products [5]. Although marginal, this borderline *p*-value suggests a meaningful tendency that may be partially explained by the high familiarity and habitual consumption of hamburgers, which can attenuate the impact of health-related attitudes on purchase intention [28]. Previous studies indicate that for widely consumed and familiar products, sensory expectations and habitual acceptance may override attitudinal drivers, reducing statistical sensitivity even when directional trends are observed [31,41]. This result may also reflect greater familiarity with or lower sensory resistance to hamburgers compared with sausages, given that hamburgers are widely consumed products and are more readily associated with functional reformulations [41].

The results presented in Table 5, together with those shown in Table 4, demonstrate an alignment between beliefs and purchase intentions, confirming that the perception of healthiness is a critical determinant of acceptance of innovative meat products [5]. However, it is also evident that the self-reported behavior of following a healthy diet does not necessarily translate into a higher purchase intention for all reformulated products. This finding suggests the presence of a “perceived healthiness paradox,” whereby preferences for healthy products may coexist with resistance related to sensory changes, price, or familiarity with food technologies [20,28].

Overall, these findings highlight the market potential for meat products with appeals to naturalness, healthiness, and sustainability, and reinforce the importance of clear communication and labeling strategies that emphasize the functional benefits of natural antioxidants, the sustainable nature of innovation, and the reformulation of food products as a safe and beneficial practice [1,4,8,11].

## 4. Conclusions

The findings of the present study demonstrate strong coherence among the dimensions of naturalness, healthiness, and sustainability. The contrasting negative and positive associations attributed, respectively, to meat products formulated with synthetic and natural antioxidants; the favorable attitudes toward naturalness, health, and sustainability in food consumption; as well as the correlations observed among these constructs and the positive influence of perceived health benefits on declared purchase behavior collectively indicate consumer openness to innovation and interest in meat products carrying these attributes. The predominance of young, female participants with higher education levels suggests that consumers most engaged with food innovation, naturalness, healthy eating, and environmental responsibility tend to be concentrated within socially connected groups with greater access to information. This profile reveals a potential market niche and an opportunity for broader acceptance of reformulated meat products. However, the restriction of the results to this specific demographic profile represents a limitation of the study, as its online nature may have favored more educated and digitally connected consumers. This limitation highlights the need for further research on consumer behavior using more diverse and representative samples to fully capture the heterogeneity of perceptions and motivations within the Brazilian population.

Overall, this study underscores the relevance of developing reformulated meat products and the importance of implementing effective communication strategies that clearly convey the innovative and beneficial nature of such reformulations. From a practical perspective, targeted labeling strategies emphasizing the valorization of agro-industrial by-products, the natural origin and functional role of antioxidants, and their contribution to environmental sustainability may enhance consumer trust and product differentiation. In addition, educational communication initiatives and transparent on-pack information may help reduce knowledge-related barriers and foster broader market adoption of reformulated meat products. These efforts may support consumer acceptance and the consolidation of these products in the market, ultimately contributing to more conscious consumption practices aligned with contemporary demands for naturalness, healthiness, and sustainability.

## Figures and Tables

**Table 1:** Sociodemographic characteristics of the participants. *n* = 422.

Variable	Category	**n**	%
Gender	Female	295	69.7
	Male	127	30.0
	Other	1	0.3
Age (years)	18–25	133	31.4
	26–35	151	35.6
	36–45	74	17.5
	46–55	45	10.6
	≥56	21	4.9
Region of residence	Southeast	373	88.6
	South	18	4.3
	Midwest	15	3.6
	Northeast	10	2.4
	North	5	1.1
Educational level	Elementary school	4	0.9
	High school	17	4.0
	Technical course	8	1.9
	Undergraduate degree	191	45.0
	Postgraduate degree	204	48.1
Household income	<1 minimum wage	30	7.1
	1–3 minimum wages	123	29.2
	3–5 minimum wages	99	23.5
	5–10 minimum wages	103	24.5
	>10 minimum wages	66	15.7

**Table 2:** Frequency of responses to statements in the attitudinal questionnaire according to the 3-point Likert agreement scale.

Statements	Likert Scale			
	I Disagree	Indifferent	I Agree	Total
QA1. I’m not afraid to eat food I have never tried before	20.4	16.1	63.5	100
QA2. I am constantly trying new and different foods	24.9	16.1	59.0	100
QA3. I follow a healthy and balanced diet	23.2	26.8	50.0	100
QA4. I would eat healthier foods even if I did not like them as much	25.1	21.3	53.6	100
QA5. I am very concerned about the healthiness of foods and care about my health	20.4	26.8	52.8	100
QA6. Consuming natural antioxidants may reduce the risk of certain diseases	6.4	18.0	75.6	100
QA7. I am willing to consume products that have a positive impact on my health	3.3	6.6	90.0	100
QA8. I am interested in consuming products that have a positive impact on my health, even if they are more expensive	1.2	18.0	66.8	100
QA9. I like to eat foods that are easy to prepare on a daily basis, even if they are less healthy	40.0	22.7	37.2	100
QA10. When I eat fast food, I do not care whether it is healthy	58.1	18.0	23.9	100
QA11. Sustainable food production is important to me	8.3	21.8	69.9	100
QA12. I am interested in purchasing sustainably produced products, even at a higher price	22.5	24.4	53.1	100
QA13. I believe it is not my responsibility to worry about sustainability in food production	71.6	12.3	16.1	100
QA14. I believe that the agribusiness sector should strive to minimize waste and environmental impacts	5.0	10.4	84.6	100
QA15. It is easy for me to purchase sustainably produced food	37.9	30.1	32.0	100
QA16. Sustainability in food production is not a priority for me	65.4	17.3	17.3	100

**Table 3:** Significant positive bivariate Pearson correlations (r ≥ 0.40, indicating moderate correlation strength) among the statements in the attitudinal questionnaire.

Statements	Pearson Correlation	Sig. (2 Extremities)	Lower	Superior
QA3–QA5	r = 0.498	<0.001	r = 0.423	r = 0.567
QA6–QA7	r = 0.436	<0.001	r = 0.355	r = 0.510
QA7–QA11	r = 0.513	<0.001	r = 0.439	r = 0.580
QA7–QA14	r = 0.572	<0.001	r = 0.504	r = 0.633
QA8–QA12	r = 0.604	<0.001	r = 0.540	r = 0.662
QA9–QA10	r = 0.431	<0.001	r = 0.350	r = 0.505
QA11–QA12	r = 0.567	<0.001	r = 0.498	r = 0.628
QA11–QA14	r = 0.438	<0.001	r = 0.357	r = 0.512

95% confidence intervals (lower and upper bounds). Estimates were calculated using Fisher’s r-to-z transformation.

**Table 4:** Contingency analysis (χ^2^) examining the relationship between purchase intention for chicken sausages and hamburgers formulated with synthetic antioxidants (sodium erythorbate) or natural antioxidants (avocado peel and seed extracts) and the belief that the consumption of natural antioxidants can reduce the risk of certain diseases (%).

Product	Consuming Natural Antioxidants May Reduce the Risk of Some Diseases	Purchase Intent			*p*
		I Wouldn’t Buy It	Maybe I Would Buy It, Maybe I Wouldn’t	I Would Buy	
	I disagree	25.9 _b_	18.5 _b_	55.6 _a_	0.029
Sausage AS	Indifferent	30.3 _b_	28.9 _a_	40.8 _b_	
	I agree	41.1 _a_	29.5 _a_	29.5 _c_	
	I disagree	33.3 _a_	18.5 _a_	48.1 _c_	0.002
Sausage AN	Indifferent	25.0 _b_	17.1 _a_	57.9 _b_	
	I agree	11.0 _c_	19.4 _a_	69.6 _a_	
Hamburger AS	I disagree	22.2 _c_	22.2 _b_	55.6 _a_	0.002
	Indifferent	28.9 _b_	30.3 _a_	40.8 _b_	
	I agree	40.8 _a_	28.7 _a_	30.6 _c_	
	I disagree	33.3 _a_	14.8 _b_	51.9 _c_	0.027
Hamburger AN	Indifferent	18.4 _b_	23.7 _a_	57.9 _b_	
	I agree	12.2 _c_	20.1 _a_	67.7 _a_	

“AS” = synthetic antioxidant; “AN” = natural antioxidant. Each subscript letter indicates a subset of categories for the statement “consumption of natural antioxidants may reduce the risk of certain diseases” whose column proportions do not differ significantly from each other at the 0.05 significance level. *n* = 422.

**Table 5:** Contingency analysis examining the relationship between purchase intention for chicken sausages and hamburgers formulated with synthetic antioxidants (SA; sodium erythorbate) or natural antioxidants (NA; avocado peel and seed extracts) and individuals’ intention to follow a healthy and balanced diet (%).

Product	Follow a Healthy and Balanced Diet	Purchase Intent			*p*
		I Wouldn’t Buy It	Maybe I Would Buy It, Maybe I Wouldn’t	I Would Buy	
Sausage AS	I disagree	19.9 _b_	20.5 _b_	59.6 _a_	0.014
	Indifferent	24.8 _a_	26.4 _b_	48.8 _b_	
	I agree	25.7 _a_	34.3 _a_	40.0 _c_	
Sausage AN	I disagree	36.5 _a_	17.5 _c_	46.0 _b_	0.024
	Indifferent	21.3 _b_	21.3 _b_	57.5 _a_	
	I agree	20.8 _b_	30.5 _a_	48.7 _b_	
Hamburger com AS	I disagree	16.9 _b_	22.1 _c_	61.0 _a_	0.005
	Indifferent	28.1 _a_	28.1 _b_	43.8 _b_	
	I agree	27.1 _a_	31.8 _a_	41.1 _b_	
Hamburger com AN	I disagree	35.5	16.1	48.4	0.068
	Indifferent	24.4	25.6	50.0	
	I agree	20.1	29.6	50.4	

“AS” = synthetic antioxidant; “AN” = natural antioxidant. Each subscript letter indicates a subset of *“healthy and balanced diet”* categories whose column proportions do not differ significantly from one another at the 0.05 significance level. *n* = 422.

## Data Availability

The original contributions presented in this study are included in the article. Further inquiries can be directed to the corresponding author.
